# Medical and Welfare Officers beliefs about post-deployment screening for mental health disorders in the UK Armed Forces: a qualitative study

**DOI:** 10.1186/s12889-015-1695-4

**Published:** 2015-04-09

**Authors:** Samantha Bull, Gursimran Thandi, Mary Keeling, Melanie Chesnokov, Neil Greenberg, Norman Jones, Roberto Rona, Stephani L Hatch

**Affiliations:** School of Psychological Sciences, University of Manchester, 2nd Floor Zochonis Building, Brunswick Street, Manchester, M13 9PL UK; Center for Innovation and Research on Veterans and Military Families (CIR), School of Social Work, University of Southern California, 1150 S. Olive Street, Suite 1400, Los Angeles, CA 90015 USA; Department of Psychological Medicine, King’s College London, Weston Education Centre, 10 Cutcombe Road, London, SE5 9RJ UK

**Keywords:** Post-traumatic stress disorder (PTSD), Alcohol misuse, Military, Thematic analysis, Secondary prevention, Health provision

## Abstract

**Background:**

This study aimed to examine currently serving United Kingdom (UK) military Medical and Welfare Officers views on the potential introduction of post-deployment screening for mental ill health.

**Methods:**

Semi-structured interviews were conducted with 21 Medical and Welfare Officers. Interview transcripts were analysed using data-driven thematic analysis.

**Results:**

Four themes were identified: positive views of screening; reliability of responses; impact on workload; and suggestions for implementation. Interviewees viewed the introduction of screening post-deployment as likely to increase awareness of mental health problems whilst also reporting that service personnel were likely to conceal their true mental health status by providing misleading responses to any screening tool. Concern over reliability of responses may provide one explanation for the reluctance of service personnel to seek help for problems, as they could feel they will not be taken seriously. Welfare Officers felt they would not have the knowledge or experience to respond to help-seeking. Although participants were concerned about potential impact on their personal workload, they indicated a desire to positively engage with the screening programme if research showed it was an effective tool to improve mental health care.

**Conclusions:**

Welfare and healthcare providers are well disposed towards a screening programme for mental health but highlight a few concerns in its implementation. In particular Welfare Officers appear to require more training in how to respond to mental ill health. Concerns about available funding and resources to respond to increased workload will need to be addressed should post-deployment screening for mental health be introduced in the UK military.

## Background

Following deployment, a number of Armed Forces personnel experience mental ill health, with symptoms ranging from post-traumatic stress disorder (PTSD) to alcohol abuse [1,2]. Whilst there are a range of mental health services available for service personnel who require them, previous findings show that many of those who experience mental ill health post-deployment do not access these services and are reluctant to seek help [3,4]. Help-seeking for mental ill health among Armed Forces personnel is lower than help-seeking for other types of health problems, and those who seek help tend to do so only once the problem has become more severe, i.e. when function is impaired or a person meets the criteria for two or more mental health problems [5,6]. This reluctance to seek help has been shown in veterans and civilians too [7]. Developing a method to encourage early uptake of mental health services could improve the response to such support and minimise the impact of mental ill health on an affected individual.

One possible method for improving uptake of mental health services is the use of mental health screening. Other military forces already use screening for mental ill health post-deployment, notably the United States (US) Department of Defense (DOD) [8,9]. This was introduced shortly after troops first deployed to Iraq in 2003 and has evolved since. Initially, screening was conducted within two weeks of service personnel returning home from deployment [10]. As it has been shown that symptoms of mental ill health may not be displayed or experienced until later on after returning from deployment [11], the screening tool was developed to contain a 3–6 month follow up component which picked up additional cases of mental ill health not identified by the earlier screen [12].

The main argument in support of post-deployment screening of military personnel for mental ill health, is that it could identify mental health problems sooner or whilst these are less severe. Early identification allows for early intervention and could potentially decrease the impact that mental ill health has on the individual [13]. A qualitative study of UK service personnel’s opinions on the potential introduction of screening found that whilst screening was considered worthwhile, lack of confidence in military health care, along with lack of trust that results would be confidential, stigmatised beliefs and concerns about impact on career would prevent service personnel from answering honestly [14]. A proposed computerised screening tool is currently being assessed by cluster Randomised Control Trial (RCT) in the UK Armed Forces. The RCT seeks to assess the suitability of such a tool and answer the question of how effective screening for mental ill health post-deployment is at identifying cases of mental ill health and encouraging help-seeking. A pilot study for the RCT included qualitative interviews exploring the views of service personnel recently returned from deployment to Afghanistan towards post-deployment screening. Whilst participants displayed positive attitudes towards screening, they also expressed a reluctance to follow health advice due to beliefs that military personnel should be seen as strong [15].

The current tool under examination in the RCT directs participants towards agencies in the military for informal support or treatment depending on the type and severity of the problem. This is likely to increase attendance to Medical Officers (MOs) and Welfare Officers (WOs) whose support for the screening programme is vital for it to have a positive impact on service personnel benefitting from the screening programme. A lack of support from military service providers may reduce the interest of serving personnel in approaching these services following tailored advice related to mental health problems. The aim of the current study is to use a qualitative methodology to examine the views of MOs and WOs on the potential introduction of a mental health screening programme post-deployment. The views of UK military health care providers have hitherto not been investigated on this topic.

## Methods

Participants were 21 members of medical and welfare staff currently serving in the UK Armed Forces; 11 MOs and 10 WOs. MOs are qualified medical doctors who have completed officer training at the Royal Military Academy Sandhurst. They complete additional training including a course on Military Psychology following this qualification. Their responsibilities include routine medical care, as well as responding to the specialist health care needs which may arise on deployment. The unit Welfare Office provides a confidential welfare support service to service men and women, families and the unit Chain of Command. The WO provides primary support to help alleviate the potential welfare issues associated with military service. WOs are usually senior non-commissioned officers or long-serving commissioned officers. One key requirement for the role is a comprehensive understanding of military service and life. A two-part WO course is currently provided centrally as well as training to Unit Welfare Office staff at various garrisons within the UK and overseas. The courses cover subjects such as child protection, domestic abuse, housing issues, bereavement and mental health. Training includes development of listening and responding skills, and understanding the role of supporting agencies.

Participants were directly recruited by researchers during visits to military units in the initial stages of the RCT of a post-deployment mental health screening tool. Informed written consent was obtained from all participants.

Interviews were conducted by one of two authors (SB and GT) with training and experience of qualitative interview techniques. One participant requested to be interviewed in person, all other participants preferred to be interviewed by telephone. Prior to commencing the interview participants were assured of the voluntary nature of their participation and confidentiality of their responses. They were also informed that their responses would not be interpreted as being indicative of the military as a whole. Interviews were recorded on a Dictaphone and transcribed by a third party. Interview length ranged from 20 to 50 minutes.

The semi-structured interview schedule comprised of nine open-ended questions; four enquired about mental health services in the UK Armed Forces as they currently stand and five focused on the proposed introduction of screening. The five questions focusing on mental health screening will be considered in the following thematic analysis. The questions aimed to gain insights into the views MOs and WOs hold about screening, and to explore perceived positives and negatives of introducing post-deployment screening, along with difficulties and workload pressures MOs and WOs think may arise due to the implementation of screening. The open-ended nature of the questions enabled participants to provide their own views on the topic, which could be explored further through prompting by researchers. The five questions were as follows:

- What do you think about post-deployment screening?
- What problems do you envisage for the provision of services if a screening programme for mental illness were implemented in the UK military?
- How would screening impact on your working life?
- If someone came to you having been advised to do so following screening, how would you respond to this person?
- Overall, how do you feel about the potential introduction of post tour screening for mental health?

Throughout the interview schedule interviewers explored both positive and negative aspects of introducing mental health screening. Where negative comments or concerns about screening were raised, interviewers prompted for suggestions to overcome these reservations.

Two pilot interviews were conducted to assess the acceptability of the questions to participants; no changes were required to the interview schedule following these pilot interviews. These pilot transcripts were included in the final analysis. The analysis was exploratory and data-driven; the researchers had no pre-conceived themes or conceptual framework. SB and GT initially analysed a random selection of eight transcripts, with regular meetings to discuss any variability in the arising themes and to generate an agreed set of common themes across the interviews. After identifying a common set of themes SB coded all transcripts and produced summaries of the key themes for each. Common patterns and divergences were then looked for across all themes from each transcript. In addition to analysing the data set as a whole, interviews and the summaries of themes for each interview were grouped according to source (either MO or WO) and common themes in each group explored so potential variations between MOs and WOs beliefs could be examined. Master themes were created by merging and connecting the common themes in the 21 transcripts. The numbers of participants in each sub group was sufficient to reach saturation, both of themes across the whole data set, and of differences between the two groups. The master themes represent all of the most commonly presented topics, as well as topics of particular importance to the matter of implementing screening. Each of these master themes contained sub themes representing different aspects or experiences described by the participants. Once the master themes had been created, SB returned to the individual transcripts to ensure these were truly representative of the beliefs of the interviewees. Simple counting was used to give an indication of the prevalence of the themes in the data [16].

At all stages during the analysis, SB and GT remained reflective and continually returned to the individual transcripts. At the time of analysis, SB was involved in the RCT of mental health screening; therefore this reflective practice was vital to ensure that the master themes and sub-themes were a true representation of the interviewees’ views and not impacted upon by her own experience of screening. This reflective approach followed recommended practice for thematic analysis [17]. Analysis was conducted by hand.

The study was approved in March 2011 by The Ministry of Defence Research Ethics Committee (Ref 187/GEN/1) and the King’s College London Psychiatry, Nursing and Midwifery Research Ethics Subcommittee (Ref PNM/10/11-112).

## Results

Four master themes emerged, each reflecting different beliefs about the potential introduction of a post-deployment screening tool: positive views of screening; reliability of responses; impact on workload; and suggestions for implementation. Each of these master themes contain sub themes as presented below. A statement of how many interviewees discussed each theme is also provided (e.g. 5/11 MOs and 6/10 WOs). The single counting procedure is intended to demonstrate the prevalence of themes in each participant group, and should not be understood as a basis for statistical generalisation to the population [16].

### Positive views of screening

This theme has two sub themes representing different positive aspects of introducing mental health screening. Firstly, participants perceived some advantages in that screening would raise the profile of mental health in the UK Armed Forces. The second sub theme was that use of a computer-based questionnaire format was seen as another positive aspect of the current screening tool being used in the RCT.

### Screening would raise mental health awareness

Interviewees felt that the introduction of a screening programme would show that the Ministry of Defence (MoD) is engaging with the issue of mental health and taking mental health care seriously. They perceived that raising the profile of mental health in this way would increase awareness of mental health issues in the military, both at an individual, and an organizational level. This was reported both by MOs and WOs (5/11 MOs and 6/10 WOs).

“I like the idea of it …to try to push and drive change I think it’s invaluable. It’s also I think a good sort of vehicle to then educate as well”. MO (19)

“I think it only gives nothing but positivity showing that there is the MoD, the Army, your organisation that there is a body out there that is concerned and working and conscious of the trauma that some bodies may have faced”. WO (10)

One WO discussed his conversations with service personnel who had taken part in the RCT:

“The feedback was ‘I never knew I was feeling like that until I answered those questions’. Must have been really provoking in their minds for them to actually say that. I’d say a good dozen guys that I spoke to were very…. They were surprised how much they’d learnt about themselves without even thinking about it so I think that’s a good sign”. WO (13)

The WO felt this feedback from service personnel provided strong support for the use of the screening tool, and showed that it would encourage open discussions of mental health issues.

### The format of the screening tool

The current format of the proposed screening tool was viewed positively; younger soldiers were viewed as being accustomed to interacting with computers and therefore would be comfortable completing the questionnaire in this way. It was believed that the current format was particularly user friendly and accessible, and would be perceived as more confidential than a face to face interview with a medical professional. This was largely reported by MOs (5/11); only one WO mentioned the user friendly format.

“It’s a more confidential way of somebody to… if they don’t want to see somebody face to face, they can fill out a questionnaire and get feedback on it. So I suppose it’s another way for them to kind of access mental health care or you know get advice to say should they be accessing mental health care”. MO (6)

“I think it’s a good idea and I think you know, the younger guys in particular, are very keen on computers and playing and clicking buttons so that will probably be seen as quite low threat”. MO (12)

### Reliability of responses

A common concern was that service personnel would find the screening tool easy to manipulate, both for masking and for exaggerating symptoms, therefore their responses would not be fully representative of their mental health state. Whilst the majority of interviewees did not express concerns about symptoms either being masked or exaggerated, this concern may impact on implementation of a screening programme and therefore was included in the list of master themes.

### Masking symptoms

A few MOs (4/11) and WOs (2/10) questioned whether the responses soldiers gave to the questions on the screening tool would be a reliable representation of their actual state of mental health. It was suggested that service personnel who did not want to admit to having mental ill health would be able to mask their symptoms by giving inaccurate responses.

“They see the tour as over, it’s done, it’s finished, let’s move on. Some of them don’t want to look back. Do people answer honestly? I don’t know”. WO (10)

“They are likely to deny, displace any significant problems they’ve got … umm because they will see it… my perception is that they will see it very much as a test and something that they can pass or fail”. MO (16)

### Malingering

Some interviewees expressed a concern that a screening tool could be manipulated to produce a false positive response as easily as a false negative. It was felt that some soldiers who currently report mental health problems do so in order to obtain extra leave, or be moved to a different unit which was particularly seen as a common problem for Germany-based units. More MOs (7/11) felt that malingering was a problem they had come across than WOs (2/10).

“Boys will be boys and they find innovative and almost constantly new ways to get out of work or, again these are often based on rumours… how to leave the service and get a medical discharge so I think some of them might think that’s a way out”. MO (19)

“Mental health is used as a big lever in a small number, but a very time consuming number of soldiers who have a number of discipline and other issues….I think I also hold the view that some of them do hide behind the mental health team and just hang on you know almost indefinitely…. They can’t be disciplined, they can’t be discharged”. MO (12)

“There are people out there that wouldn’t be honest and would probably again try and hide behind it and you know tick the boxes that they are identifying themselves as being at risk and therefore you know you are never going to get rid of that….. those type of people who are going to play the system”. WO (17)

### Impact on workload

The impact that screening may have on a MO or WOs workload was the most consistent theme. Perhaps unsurprisingly, participants were keen to discuss how their working lives may be affected by the screening process. Sub themes within this theme included staffing levels, funds and resources, the role of the WO and interviewees feeling happy about the potential increase in workload should it have positive impact on the mental health of service personnel.

### Staffing levels

The numbers of staff needed both for running the screening itself, and then to cope with a potential increase in service use were seen as important considerations. This was highlighted by more MOs (7/11) than WOs (2/11).

“That would be manpower intensive and then it’s going to have to be… we are going to prioritise even more because then you are going to have to look at giving priority to ops so if the screening tool brings somebody up then they are going to have to be seen I am sure there will be parameters for that”. WO (3)

“You know we’re obviously… if we’re going to signpost more people then we’re probably going to need more people to deal with you know the people that we’re identifying who may need help”. WO (17)

### Funds and resources

The question of finances available for health services came up in some MO interviews (5/11) and in a few WO interviews (2/10). One MO pointed out that there are only limited funds available, which need to be shared between mental health and physical health services.

“For us to justify the not inconsiderable expense of screening the entire Armed Forces every time they go on tour” MO (16)

“And whether it’s going to take… umm… resources away from other aspects of military health. I mean if it… both be that physical injuries as well as mental injuries and I think that you know there is a finite amount of cash isn’t there? MO (18)

### Welfare Officer Role

WOs were particularly concerned about their role in screening and its impact on their workload. Half of the WOs interviewed (5/10) expressed that there was some confusion over their role, and that in the case of mental health they serve as a signpost to appropriate services, therefore it would be more effective to send people directly to the MO. Some WOs (4/10) suggested that additional training in mental health and how to respond to service personnel post-screening would assist WOs to engage with the process more effectively.

“You know and it says on the screening ‘go and seek advice from the Welfare Officer’. What do I tell them?” WO (4)

“I think if somebody has got an issue and it is highlighted they need to go to somebody who is qualified and prepared to accept them and that needs to be resourced rather than just giving it to the Welfare Officer”. WO (2)

### Happy to have an increase in workload

Whilst all participants (21/21) were concerned about the impact screening would have on their workload, many stated that if screening had a proven positive impact on access to services, they would be happy to take on this additional work. This finding was consistent across MO and WOs responses (5/11 MOs, 6/10 WOs), despite many WOs saying they felt it would be more effective for the screening tool to direct service personnel straight to the MO.

“Yeah that’s what I’m here for ultimately”. WO (8)

“I think that’s an unfair question actually because it’s not whether it increases… it improves my working life, it’s whether it improves the services for patients isn’t it? And I think that… I can see it giving me extra work but that doesn’t mean it’s a bad thing”. MO (18)

### Suggestions for implementation

Both MOs and WOs had a number of suggestions for ways in which the screening tool could be implemented in order to achieve a high level of efficacy. Sub themes within this master theme were confidentiality, timing and augmentees (individuals who deploy with a different unit from their own) and reservists.

### Confidentiality of results

There was a lot of divergence within this sub theme, with eight interviewees (4/11 MOs and 4/10 WOs) suggesting that results of the screening tool should be made available to a medical professional or the unit WO, whilst three interviewees (2/11 MOs and one WO) felt strongly that the results should be absolutely confidential. The justification for making results available to somebody was that keeping results confidential would not impact on help-seeking levels; service personnel would continue to avoid admitting to a problem.

“I understand medical confidentiality and all that. If we could screen and use that information… it would probably by more effective because like you say if a soldier says ‘no, I’m not interested’ but is a high risk then the chain of command and the unit may not have picked up on that yet”. WO (8)

“If you are going to do the screening then ultimately somebody is going to have to have ownership of it, and if it is a sort of validated screening we are doing then, I think somebody will have to get the results otherwise what is the point of doing it”. MO (1)

When questioned further on this issue, all participants who expressed this view also stated that they felt service personnel would be more likely to mask any symptoms they were experiencing should the results not be confidential.

“The beauty of an anonymous set up is that they can literally relax and put down what they feel”. MO (9)

### Timing of screening

All participants (21/21) felt that this would be an important and challenging factor in the decision to introduce a screening programme. Screening would need to take place at an optimum time to maximise early detection of symptoms, and detect the majority of cases, but also a time that would not impact on the current post-deployment programme.

“So it would have to be done at a time which clinically… err… would pick up important things… err… in other words you couldn’t leave it for too long after theatre but at the same time it would have to be done… where they are all still together I would suggest”. MO (9)

The timing of when the questionnaire is done has got to be done to suit the unit, when the unit is potentially not as busy. A suitable time after the tour because if you do it directly after the tour a lot of them go “no I haven’t got a problem” and actually the problem then bubbles up a few months later”. WO (4)

### Augmentees and reservists

These groups were mentioned by a few interviewees (2/11 MOs and 2/10 WOs as the service personnel most in need of improved post-deployment care. After initial decompression augmentees usually rejoin their usual unit where they do not have the support of the peers they deployed with. Reservists return to their civilian lives where they do not have the support of the peers they deployed with who can best understand their experiences. Participants suggested that screening would have the most positive impact on this subset of the UK military. Although only endorsed by a relatively small number of participants, to those who did raise this point it seemed a pertinent issue. Moreover as this is particularly relevant to screening it was included in the list of themes.

“Where perhaps the screening would be more useful is where you’ve got more diffused units, you’ve got individual augmentees, and I know in the past members of the reservists have been more vulnerable here”. MO (16)

“My biggest concern is with mental health screening and post-operational tour leave… umm… even the six to twelve week briefing, the reservists and augmentees have all gone”. WO (10)

Overall interviewees expressed a willingness to engage with mental health screening should it be implemented in the UK Armed Forces, and also felt that the presence of such a programme would raise the profile of mental health. However, concerns about reliability of responses, the availability of resources and impact of increased workload on medical and welfare services would need to be addressed should the tool be introduced as part of the post-deployment programme.

## Discussion

Four master themes on the potential introduction of post-deployment mental health screening were identified using thematic analysis [16]: positives of screening, reliability of responses, impact on workload and suggestions for implementation.

Interviewees spoke positively about the potential for a screening tool to raise the profile of mental health and facilitate open discussions about mental health between service personnel. This is a benefit of screening which is applicable to both military and civilian populations. It is possible that the process of taking part in mental health screening may cause an individual to consider their own mental health and then discuss the issue more freely with their peers. The format of the current screening tool as a computer based questionnaire was seen as appropriate and non-threatening. A recent study of a self-administered computer based screening tool for mood disorders in a primary care population found this self-report method to be more accurate than GP interviews at detecting current mood disorder, suggesting that non-military populations may also find this method more appealing [18].

A number of interviewees were concerned that service personnel would not respond reliably to the questions in the screening tool; it was suggested that service personnel may mask symptoms of mental ill-health or provide misleading responses creating false-positive results. The issue of service personnel malingering is not one that has been well investigated in the UK Armed Forces thus far. Reporting of fictitious symptoms has been reported in soldiers serving in US Armed Forces [19]. Analysis of US veterans claiming compensation for PTSD has indicated that financial incentives may influence exaggeration of symptoms in US military personnel [20,21], but the reasons for over reporting symptoms in US Forces may not be applicable to UK Armed Forces due to differences in availability of free health care and compensation schemes.

Many interviewees suggested that confidentiality and decisions surrounding whether screening results should be shared with medical staff were potentially problematic considerations. Some WOs argued in favour of having access to results because they wished to either approach the individual with potential mental health difficulties, or “keep an eye” on that person. There is considerable evidence that suggests a screening tool should remain anonymous to encourage honesty in responses. Perception of confidentiality has a measurable impact on responses to mental health questions, [22,23] and the qualitative study of service personnel views on screening reported the confidentiality of results as a positive aspect of the tool [15]. Perceived confidentiality has also been found to increase acceptability of mental health screening programmes in paediatric care [24,25].

Concerns about the practicalities of implementing screening were raised. These concerns included a perceived need to increase staffing levels to respond to increased demand for services and also whether appropriate funding and resources would be available to respond to this increased demand. For any screening programme these issues would need to be considered, weighing up the benefits of increased awareness of mental health and possibility of early intervention against the costs of providing screening and services to those who need it. A review of screening programmes for depression in primary care settings found that relevant staff assistance being in place was essential if a screening programme was going to improve depression outcomes [26].

Many WOs felt that it was inappropriate for the screening programme to advise personnel to visit them as they do not have the knowledge to properly advise personnel with mental ill-health. The current screening tool being assessed in the RCT only advises service personnel to visit the WO if their symptoms are mild, as defined by scoring on or above the threshold for depression, anxiety, PTSD or alcohol misuse; more severe symptoms (scoring higher on the screening measures) would result in personnel being advised to visit the MO. Interviewees were aware of this, but WOs still felt that they would not be the best person for giving mental health advice. This common feeling of not being able to assist with mild mental health problems may point to a need for more in-depth training for WOs, or alternatively to a need to clarify the role of the WO with relation to mental health care in the UK Armed Forces. Service personnel may feel more comfortable approaching a WO for informal advice than a medical professional; therefore there would be value in ensuring WOs felt prepared to respond to questions about mental ill health.

Interviewees had a number of suggestions for the successful implementation of screening in the UK Armed Forces. It was felt that the timing of screening would need to be considered carefully in order to provide the most useful tool in terms of a clinically relevant time frame, taking into consideration the fact that some symptoms of mental ill health may not be present until later on following return from deployment [11]. The ease of fitting a screening programme into an already busy post-deployment schedule would also need to be considered. Augmentees and reservists were a key concern; it was felt that this population would benefit most from post-deployment screening. Augmentees do not appear to have more problems post-deployment, however, reservists who deployed to Iraq have been shown to have increased risk of PTSD post-deployment and 5 years on [27,28]. Reservists have reported feeling unsupported by the military following deployment, therefore involving this group in mental health screening may increase their feeling of being supported by the military [29]. The suggestion that screening may be of most benefit to augmentees and reservists is interesting as they are not currently being assessed by the screening RCT; this population may require further investigation. Planned changes to the structure of the UK Armed Forces will result in a more equal ratio of reservist to regular personnel being deployed; therefore it would be important that any screening tool is able to provide advice for this subset of the military.

A strength of the study was the qualitative method used, specifically semi-structured interviews. This allowed for exploration of personal views held by medical and welfare professionals on the topic of the potential introduction of screening, without being constrained by pre-defined response options. This led to the emergence of topics and issues not expressly referred to in the original interview schedule. For example, interviewees were not specifically asked about augmentees and reservists, yet the importance of the issue was able to emerge due to the nature of this method. One limitation of the study is the social desirability component of the question “*If someone came to you having been advised to do so following screening, how would you respond to this person?*” Although participants had been assured that their responses were confidential, there may have been some perceived pressure to respond to this question in a positive way. The variety in experience of those interviewed for the study may be a weakness, for example Welfare Officers can come into their role from a number of different backgrounds, with differing levels of exposure to mental ill health prior to the role. These differences in experience of mental ill health may impact on how interviewees responded to the idea of introducing mental health screening. Due to the necessary timings of interviews based on interviewees’ availability, not all interviewees had personally experienced the screening programme prior to completing their interview. All interviewees were knowledgeable about the screening tool, the aims of the RCT and belonged to a unit involved in the trial. This personal involvement with the trial may have encouraged interviewees to think of its impact in terms of their own unit and how they feel they would engage with it.

The results of this thematic analysis raise important considerations for the MoD and any service looking to implement a mental health screening programme. Participants expressed concern that service personnel would give unreliable responses to the questions in the screening tool, producing either false-positive or false-negative responses. In addition to offering reassurance on confidentiality of responses, it may be beneficial for education on the importance of mental health screening to be provided for participants alongside any screening tool, to encourage reliable responses. Concerns about staffing and financial resources which may be further strained by the introduction of screening would need to be assessed before such a programme is introduced. The issue of WOs not feeling prepared to respond to service personnel with mental ill health may be an area for consideration by the Armed Forces; more training in this area may be advisable.

Despite concerns about the impact of mental health screening on workload both for MOs and WOs, the majority of interviewees felt they would still engage with the screening and many said they would be happy to see an increase in their work load if it enabled earlier interventions and help-seeking for mental health problems. This suggests medical and welfare personnel would positively engage with an effective screening tool for mental health problems should it be introduced to the UK Armed Forces in the future.

## Conclusions

Military medical and welfare service providers are well-disposed towards a screening programme for mental health. Concerns over funding and resources would need to be considered should such a programme be introduced, and the suggestion that service personnel may not provide reliable answers would need to be addressed. The role of the WO in mental health services may need to be clarified, or further training in mental health may be required for service personnel in this role.

## Abbreviations

PTSD: Post-traumatic stress disorder
DOD: Department of Defense
MoD: Ministry of Defence
MO: Medical Officer
WO: Welfare Officer
RCT: Randomised Controlled Trial
UK: United Kingdom
US: United States
